# Madame Boivin on Polypus of the Uterus and Pregnancy

**Published:** 1829-10-01

**Authors:** 


### MAISON ROYALE DE SANTE.

### POLYPUS OF THE UTERUS AND PREGNANCY.*

The above combination is singled out as the subject of an interesting report by—*a lady*, MADAME BOIVIN. Ah, Sir Anthony Carlisle, how would your heart leap with joy, could you see such obstetric *blues* in merry England, or preside over a college of literary midwives *not* men! How would decency be screened if the *harem* of generation and fœtation were forbidden ground to all who are not arrayed in petticoats, and the mysteries of midwifery only to be descanted on by females! Seriously, however, there appears to be something repulsive in the idea of women writing on such subjects as impregnation, or relating dissections with perfect sang froid, something which would seem to sin against decency and feeling. But after all, men's sentiments on these points are conventional rather than established on any certain principle, and what passes for modesty and decorum here, is looked on with dismay by the *faithful* or the prudes of Constantinople. If our Gallic neighbours tolerate and encourage female practitioners and writers in midwifery, surely we have no business to complain. We remember reading of a cardinal, who, when the pious sisters of a certain nunnery complained that they were scandalized by the posterior view of a naked marble

* *Journ. Hebd. No.* 44.

god, erected in his holiness's garden, recommended them to look another way! If any of *our* pious brethren are shocked at French ladies publishing on pregnancy we would simply repeat the cardinal's advice, but for our own parts we shall make use of the information they afford.

The combination of polypus uteri with pregnancy is not uncommon, and we lately adverted to one or two cases in a review of a paper of Dr. Macfarlane's on the subject of polypus. Madame Boivin has related three cases, the first of them fatal, which are short and deserving of attention.

*Case* 1. Mademoiselle Anne Lem. æt. 22, a semstress of the rue St. Honoré, was brought to the Maison de Santé, on the 16th of August, 1822, fifteen hours after delivery at the full time, of a living child, her third. The infant was removed five hours afterwards to be deposited at the Foundling Hospital, when the mother who had been previously doing well, fell into convulsions and delirium. On admission her state was hopeless, and thirty hours after her accouchement she died.

*Sectio Cadaveris.* There was purulent matter on the surface of the cerebellum, and the liver was tuberculated, but passing over other matters briefly, let us look to the condition of the uterus. The neck of the organ which was quite collapsed was occupied by a polypus, three inches in length by two and a half in breadth, attached by a broad pedicle to the posterior part of the internal orifice of the uterus. The tumour was covered half way up by a thin whitish membrane, continuous with that which still lined a part of the uterine cavity. The placenta attached to the posterior wall of the uterus, had also an attachment to the superior and anterior portion of the polypus. The structure of the latter was pulpy, reddish, and like the fœtal brain, but more consistent.

As Madame Boivin remarks, it is evident from the placental attachment to the polypus, that its origin was nearly coincident with, if not antecedent to, pregnancy. Notwithstanding this the patient went her full time and was delivered of a living child.

*Case* 2. Madame P. R.———, wife of a linen-draper at Louviers, consulted Mad. Boivin for bloody discharge from the vagina, to which she had been subject for many years. She had met with five or six successive abortions, and her first and only living child, was rickety. On examination, a polypus about the size of a plum was felt at the cervix uteri, which had descended very low; its substance was soft, and the finger could be passed along the pedicle into the uterine cavity. Notwithstanding Madame Boivin's urgent recommendations to the patient to submit to an operation, she refused until another year and an eighth abortion in the third month had occurred, with increase of the bloody discharge. Then and not till then, she allowed M. Dupuytren to apply the ligature, a tuberculated state of the cervix uteri ensued, and in the course of two years more the patient died.

*Case* 3. In April, 1820, a lady residing at Saint Germain-en-Laye, applied to Mad. Boivin on account of an amenorrhœa, which had lasted since her third and last accouchement, seven years previously. The labour, from her husband's account, had been natural, and delivery prompt. When the accoucheur was leaving the room, the patient complained of weakness and of something large escaping from the parts, which proved to be a polypus, as large as the fist. The accoucheur applied no ligature, but tore the tumour from its attachment to the uterus, by twisting the pedicle, an operation attended by violent pain at the time, and followed by inflammation of the parts, which kept the patient in her bed for several months. The menses never afterwards appeared, but were replaced by a nearly constant flow of white fluid, which reduced the patient to a great degree.

At the time of her application, the expression was that of extreme exhaustion, there was constant lassitude and dragging at the stomach, and the patient complained of a perfect indifference to conjugal intercourse; in fact, she said that since her last accouchement, she seemed to have lost her sex. On examination per vaginam, nothing particular was noticed, except that the cervix seemed low, and its orifice very open; the uterus appeared to have preserved its natural volume.

Madame Boivin asks very reasonably, if the suppression of the catamenia in this female, only twenty seven years of age, is to be referred to the inflammation of the uterus, ending in adhesion of its sides? She also adverts before closing the report to the following curious facts; the daughter of the last patient has distortion of the spine, the son of the second was rickety, and another lady, of Madame Boivin's acquaintance, who had been operated on for polypus uteri three months after confinement, has also given birth to a deformed daughter. The coincidences are curious.

